# Response to a Letter to the Editor from Katherine Rich

**DOI:** 10.3390/nu7075264

**Published:** 2015-07-20

**Authors:** Helen Eyles, Cliona Ni Mhurchu, Delvina Gorton, Yannan Jiang, David Monro

**Affiliations:** 1 National Institute for Health Innovation, The University of Auckland, Private Bag 92019, Auckland 1142, New Zealand; E-Mails: c.nimhurchu@auckland.ac.nz (C.N.M.); y.jiang@auckland.ac.nz (Y.J.); 2 Epidemiology and Biostatistics, The University of Auckland, Private Bag 92019, Auckland 1142, New Zealand; 3 Heart Foundation of New Zealand, P.O. Box 17160, Greenlane, Auckland 1546, New Zealand; E-Mails: info@heartfoundation.org.nz (D.G.); davem@heartfoundation.org.nz (D.M.)

A letter to *Nutrients* from the Chief Executive of the New Zealand Food and Grocery Council [1] questioned the validity of methods and findings reported in our recent paper, “Changes in the Sodium Content of New Zealand Processed Foods: 2003–2013” [2]. We welcome informed discussion on sodium reduction, an issue of fundamental importance to the health of New Zealanders and a key target in the World Health Organization Global Action Plan to reduce premature mortality from non-communicable diseases (NCDs) [3]. However, we disagree with the author’s assertion that significant changes in the sodium content of packaged foods in New Zealand were masked by our analysis methods; we maintain that our methods were scientifically sound and our conclusion was justified. The author makes three main assertions in her letter; here we refute each in turn.

## 1. No Measure of Consumption was Included

Reduction in population sodium consumption is pivotal to reducing the burden of NCDs. However, the stated aim of our analysis was to examine 10-year (2003 to 2013) changes in the sodium content of key processed foods in New Zealand. Whilst food availability and purchases are key determinants of consumption [4,5], we were careful not to extrapolate our findings to consumption as can be seen from the discussion in our published paper. It is important to note however that data from national urinary sodium excretion surveys show there has been no change in average sodium intake of New Zealand adults since the 1980s [6,7]. Furthermore, the unreferenced assertion that “*a weighted average…….would tend to show quite different reductions*” is not supported by data from the UK where percent reductions in sodium content of processed foods following implementation of their national salt reduction programme were very similar, regardless of whether or not they were weighted by sales [8].

## 2. Products on Shelves in 2003 were Different to 2013

The author asserts that our analysis of changes over time was problematic because the type and number of products available on supermarket shelves was not the same in both years. Moreover, she suggests that newer products coming onto the market during that time period might actually be lower in sodium. We assessed this comprehensively by undertaking two analyses reported in the paper: An analysis of paired/matched products over time using appropriate significance testing, and another analysis of all products available for sale at each time point (2003 and 2013). The paired/matched analysis revealed no significant difference in sodium content of 182 matched products (−56 (122) mg/100 g; *p* = 0.22), and the unpaired analysis also revealed no difference in sodium content of all products over time (436 *vs*. 433 mg/100 g respectively). Both analyses demonstrate empirically that new products that came onto the market between 2003 and 2013 and included in our analysis could not have been lower in sodium content.

## 3. No Mention is Made of Significant Projects Undertaken by New Zealand Food Manufacturers to Reduce Sodium

It was not our aim to profile New Zealand food manufacturers who have undertaken sodium reduction programmes but rather to assess change in the sodium content of processed foods available for sale in New Zealand over a 10-year period. We did however discuss reformulation efforts and programmes in a general sense. Our analysis showed no significant change in the average sodium content of total packaged products assessed, however there have been worthy efforts by some food categories, such as bread and breakfast cereals.

Finally, the author makes a factual error in stating that data presented in Table 1 showed that “*more than 75% of the items examined exhibited a decline in sodium levels..*.” Table 1 describes mean sodium content, not the number of individual products which have been reformulated, and the percentages reported refer to the number of foods in each category as a proportion of the total number of products included in the analysis.

## 4. Conclusions

In summary, we maintain that the methods of our published paper were scientifically sound and our conclusion was justified. There has been modest progress with sodium reduction in some food categories [5,8]; however, much greater action is needed across the entire food supply if New Zealand is to meet its global commitment to reducing population salt intake by 30% by 2025.

## Appendix

### Footnote from the Editors-in-Chief:

Nutrients does not, as a rule, encourage debate on the content of its publications via Letters to the Editor. We made an exception in this case, however, by publishing a Letter to the Editor from Katherine Rich, representing New Zealand food manufacturers [1]. Her letter challenged the inference in a recent paper by Monro *et al.* [2] that too little has been done to lower the sodium content of foods in New Zealand. The response above from Eyles *et al*. to Rich’s Letter concludes the exchange, which reminds us that the challenge of achieving international recommendations on reduction of sodium intake is multisectorial; the responsibility must be shared with government policy makers, educators, food manufacturers and retailers, the catering industry and ultimately with consumers, who can choose to eat available foods with high or low sodium content.
